# General and Food-Specific Inhibitory Control As Moderators of the Effects of the Impulsive Systems on Food Choices

**DOI:** 10.3389/fpsyg.2017.00802

**Published:** 2017-05-24

**Authors:** Xuemeng Zhang, Shuaiyu Chen, Hong Chen, Yan Gu, Wenjian Xu

**Affiliations:** ^1^Key Laboratory of Cognition and Personality (Ministry of Education), Southwest UniversityChongqing, China; ^2^School of Psychology, Southwest UniversityChongqing, China

**Keywords:** restrained eaters, inhibitory control, impulsive system, food choice, eye movement

## Abstract

The present study aimed to extend the application of the reflective-impulsive model to restrained eating and explore the effect of automatic attention (impulsive system) on food choices. Furthermore, we examined the moderating effects of general inhibitory control (G-IC) and food-specific inhibitory control (F-IC) on successful and unsuccessful restrained eaters (US-REs). Automatic attention was measured using “the EyeLink 1000,” which tracked eye movements during the process of making food choices, and G-IC and F-IC were measured using the Stop-Signal Task. The results showed that food choices were related to automatic attention and that G-IC and F-IC moderated the predictive relationship between automatic attention and food choices. Furthermore, among successful restrained eaters (S-REs), automatic attention to high caloric foods did not predict food choices, regardless of whether G-IC or F-IC was high or low. Whereas food choice was positively correlated with automatic attention among US-REs with poor F-IC, this pattern was not observed in those with poor G-IC. In conclusion, the S-REs had more effective self-management skills and their food choices were affected less by automatic attention and inhibitory control. Unsuccessful restrained eating was associated with poor F-IC (not G-IC) and greater automatic attention to high caloric foods. Thus, clinical interventions should focus on enhancing F-IC, not G-IC, and on reducing automatic attention to high caloric foods.

## Introduction

People in developed countries have a myriad of opportunities to eat. Food, especially highly palatable food, is readily available in almost every setting during the day (Thornton et al., 2013). The modern obesogenic environment has resulted in an increasing number of people who attempt to control their weight. People who adhere to an intentional, sustained restriction of caloric intake to lose or maintain body weight are referred to as REs (Herman and Mack, 1975). However, some REs are often unsuccessful in controlling their weight, continuing to choose and eat HC foods (Mann et al., 2007). Many explanations have been proposed for problems regulating eating behaviors. Often, eating behavior seems to be governed not only by goal-directed behavior, but also by automatic reactions, as defined in the reflective-impulsive model (Strack and Deutsch, 2004; Hofmann et al., 2007, 2008, 2009b, 2011; Kakoschke et al., 2015). Thus, the present study aimed to clarify some of the explanations for these observations.

The impulsive system guides behavior through associative links (i.e., implicit preferences for food cues) and motivational orientations (i.e., automatic attention to food cues); this is an automatic process, which is fast, implicit, and effortless (Strack and Deutsch, 2004; Hofmann et al., 2007, 2008, 2009b; Kakoschke et al., 2015). Hofmann et al. (2008) suggest that an implicit measurement tool is required to measure an impulsive system effectively. For example, implicit preferences can be measured using the Implicit Association Test and automatic attention can be measured by tracking eye-movements to minimize interference from consciously controlled processing. Studies have shown that a stronger impulsive system that includes attention to food and an implicit preference for HC foods is associated with unhealthy eating behaviors (Houben et al., 2010; Werthmann et al., 2011; Field et al., 2016). In particular, studies reviewed in our literature search indicate that the impact of the impulsive system (implicit preference) on eating behavior depends on inhibitory control.

Previous experiments have found that participants with a strong implicit preference or approach bias for snack foods, combined with low inhibitory control, gained the most weight (Nederkoorn et al., 2010) and consumed the most snack foods (Kakoschke et al., 2015). Furthermore, a study by Hofmann et al. (2009a) identified G-IC as a moderator of the relationship between implicit preferences and eating behavior, with high inhibitory control decreasing the influence of implicit preferences on eating behavior. Similarly, other research has found that the effect of the impulsive system was relatively strong when food-related self-control was weak (Honkanen et al., 2012). The results of a recent study indicate that the trait of self-control ability moderates the prediction of implicit preferences on eating behavior (Wang et al., 2015). In that study, implicit preferences predicted chocolate consumption among participants with low trait self-control, but the predictive power of the implicit preferences disappeared in participants with high levels of trait self-control. These results have consistently demonstrated that higher inhibitory control buffers the effect of the impulsive system on eating behavior, whereas lower inhibitory control induces the opposite pattern.

In the present study, we aimed to advance knowledge in several areas of inquiry. First, prior studies have focused on the role of implicit preferences in impulsive systems instead of automatic attention. The important role of attentional bias in eating behavior has been reported in the research literature. Specifically, REs were found to have an attentional bias (i.e., more attention) toward HC foods compared with LC foods and neutral stimuli, which was associated with more food intake (Hollitt et al., 2010; Meule et al., 2012b; Neimeijer et al., 2013; Kemps et al., 2014; Werthmann et al., 2014). However, there are two kinds of attentional mechanisms: bottom-up and top-down mechanisms. The former, which is involved in the automatic processing of the impulsive system, unconsciously prioritizes the information that is to be noticed (LaBerge, 2002; Knudsen, 2007), such as food cues, which can lead to excessive eating. Therefore, we aimed to explore the effects of automatic attention (impulsive systems) on eating behavior. Automatic attention has been assessed by tracking eye movements during the process of making food choices. During the decision-making process, numerous cognitive activities underlying food choices are not manifested before an eventual behavioral outcome. Therefore, tracking automatic attention by observing the number of initial direct gazes during the decision-making process should help us understand the causes of unhealthy dietary behavior.

Studies have also found that poor inhibitory control leads to excessive consumption of HC food (Guerrieri et al., 2009; Houben, 2011; Hall et al., 2014), which might be caused by the enhancement of the effect of impulsive systems on eating behavior through poor inhibitory control (Hofmann et al., 2009a; Nederkoorn et al., 2010; Kakoschke et al., 2015; Wang et al., 2015). Hence, we also examined the moderating effect of inhibitory control on impulsive systems. However, most studies on this topic have tested inhibitory control in general, but not inhibitory control related to food. Research findings indicate that excessive eating is related to poor inhibitory control that is food-specific, rather than G-IC (Batterink et al., 2010; Nederkoorn et al., 2012; Houben et al., 2014). Only Honkanen et al. (2012) measured food-specific self-control, but their data were collected using self-report measures. The present study, however, used the Stop Signal Task, which is a more implicit and reliable method of measuring G-IC and F-IC, in order to clarify which type of inhibitory control would have a moderating effect and which effect would be greater. Finally, studies have rarely investigated subgroups of REs, such as S-REs and US-REs. Previous studies have only found an association between US-REs and poor inhibitory control (Jansen et al., 2009; Kong et al., 2015) and a greater attentional bias toward HC foods (Zhang et al., 2016) compared to S-REs. The mechanisms underlying unsuccessful restrained eating remain unclear. Therefore, it was necessary for us to explore the cognitive processes of REs during decision making related to food choices, to gain a better understanding of the mechanisms underlying the success and failure of dietary restriction in order to promote healthful eating.

The current study explored the effects of automatic attention (the impulsive system) on food choices, and examined the moderating effects of G-IC and F-IC among S-REs and US-REs. We aimed to answer the following question: Which type of inhibitory control moderates the relationship between impulsive systems and food choices? The results of the study were intended to clarify the reasons for successful and unsuccessful restrained eating. As reported in previous studies, S-REs have greater restraint, a tendency for lower consumption, and more effective self-management strategies, compared to US-REs (Fishbach et al., 2003; Van Strien and Ouwens, 2007; Keller and Hartmann, 2016). Hence, we hypothesized that S-REs would not be affected by automatic attention (the impulsive system), regardless of whether their G-IC or F-IC were higher or lower. Lower G-IC and F-IC among the US-REs were expected to increase their automatic attention (the impulsive system) to their food choices, and higher G-IC and F-IC were expected to have the opposite effect.

## Materials and Methods

### Participants

The participants were 64 female undergraduate students. The inclusion criterion for participation was a score higher than 3 on the Restrained Eating subscale of the Dutch Eating Behavior Questionnaire (DEBQ) (Van Strien and Ouwens, 2007; Kong et al., 2015). Participants with scores on the Perceived Self-Regulatory Success in Dieting Scale (PSRS) that were above average were classified as S-REs, and those with below-average scores were classified as US-REs (Fishbach et al., 2003; Meule et al., 2012a; van Koningsbruggen et al., 2013; Nguyen and Polivy, 2014). Additional criteria for inclusion in the study were weight within the normal range (BMI between 18.5 and 25 kg/m^2^) and right-handedness. Similar to a previous study, we excluded participants who followed a medically prescribed diet in the 6 months prior to the study and women with potential biases because of preferences for vegetarian foods (van der Laan et al., 2014). The experiment was approved by the Southwest University Human Ethics Committee and was conducted in accordance with the guidelines of the Declaration of Helsinki. Written informed consent was obtained from the participants prior to the study’s commencement.

### Procedures

Prior to the experiment, 400 questionnaires were distributed to students enrolled at Southwest University for the purpose of selecting participants, and 371 questionnaires were returned. The final sample consisted of 64 participants who were selected using the study’s inclusion criteria.

The study consisted of two parts: a preliminary experiment and a formal experiment. During the preliminary experiment, participants evaluated the expected tastiness and perceived energy content of the food stimuli on a 9-point scale ranging from 1 = tasteless/very LC to 9 = very tasty/very HC. The food stimuli consisted of 50 HC food pictures and 50 LC food pictures. All the selected food stimuli had a tastiness rating of 4 or higher to avoid forcing participants to choose food for which they had a strong dislike. The energy content of the LC foods was below 4 points and the energy content of the HC foods was above 6 points. One week later, we conducted the formal experiment. Participants were prohibited from eating or drinking anything (except water) for at least 2 h before the second study to stimulate their craving for food. Upon their arrival, participants were told that the study was a survey about food preferences. First, they were asked to rate their hunger on a visual analog scale, which ranged from 0% (not hungry) to 100% (very hungry). Next, they performed the Stop-Signal Task (SST), which measures G-IC and F-IC. Afterward, they completed a food-choice task that required them to choose one food they wanted to eat when presented with an HC and an LC food. During the food-choice task, eye movements were recorded using the EyeLink 1000 (SR Research, Mississauga, ON, Canada), to measure participants’ automatic attention to food.

### Measures

#### Restrained Eating Subscale of the Eating Behavior Questionnaire (DEBQ)

Participants’ restraint standards were assessed using the Restrained Eating Subscale of the DEBQ (Van Strien et al., 1986). The 10 items on the instrument (e.g., “When you put on weight, do you eat less than you usually do?”) are rated on a 5-pointscale (1 = never, 2 = seldom, 3 = sometimes, 4 = often, 5 = very often). The mean score of the scale represents dietary restraint. Participants with high scores are concerned about their weight and controlling their food intake.

#### Perceived Self-Regulatory Success in Dieting Scale (PSRS)

The REs’ success was measured using the PSRS, which requires participants to rate 3 items on a on a 7-point scale. The 3 items measure how successful the respondents have been in (1) losing weight, (2) monitoring their weight (e.g., 1 = very unsuccessful, 7 = very successful), and (3) how difficult they have found it to stay in shape (e.g., 1 = very easy, 7 = very difficult), the last item is reverse coded. A higher mean score indicates a higher level of success at restrained eating (Fishbach et al., 2003; Meule et al., 2012a; van Koningsbruggen et al., 2013; Nguyen and Polivy, 2014).

#### Stop-Signal Task

The SST involves two concurrent trials: A go trial, which is a choice-reaction time task, and a stop trial, which involves inhibiting responses to the go task (Logan et al., 1997). We adopted two variants of the SST based on a previous study (Houben et al., 2014): the first test measured G-IC, and the second measured F-IC. In both SST tasks, a fixation cross was presented first for 1,000 ms and the go stimuli were subsequently presented for 1,000 ms. In the general SST, the go stimuli were the left arrows or right arrows and participants were instructed to press “F” on a computer keyboard for the left arrow and “J” for the right arrow as fast as possible. In the food-specific SST, the go stimuli included pictures of HC foods. Participants were requested to press “F” on the keyboard if the food picture appeared on the left side of the screen and “J” if it was on the right side of the screen. In both SSTs, 25% of the trials presented a visual stop signal (×) after the go trial and participants were instructed not to respond to the go stimuli in such cases. The stop-signal delay (SSD) was initially set for 250 ms and was dynamically altered after each trial by a tracking procedure to enable participants to achieve correct inhibition in 50% of the stop trials. If participants successfully inhibited their response, the go-stop delay was increased by 50 ms. If they did not inhibit their response, the go-stop delay was decreased by 50 ms. Both of the SST variants consisted of one practice block without stop signals (16 trials) and two test blocks with stop signals (each block had 56 trials). The stop signal reaction time (SSRT) reflects inhibitory control (Mean SSRT = Mean Go RT-Mean SSD). Higher SSRTs indicate lower inhibitory control.

#### Eye-Movement Tracking during the Food-Choice Task

In each trial, a HC and LC food were shown side by side on a screen and participants had 3,000 ms to choose one of the two foods they would most like to eat. If they chose the food on the left side of the screen, they pressed the “F” key, and if they chose the food on the right side, they pressed the “J” key; the task was preceded by a 500 ms fixation cross. After participants made their choice, the screen was blank for 500 ms. In the food-choice task, participants made a total of 100 choices (the numbers of HC and LC foods presented on the left and right sides of the screen were balanced). At the end of the task, the percentage of participants who chose the HC foods was calculated. The participants were not told that their choices were always between pairs of HC and LC foods (van der Laan et al., 2014).

To investigate participants’ automatic attention during the food-choice task, we used the EyeLink1000. Evidence has revealed that eye movements are guided by attention (Kowler, 1995); thus, we recorded the number of initial direct gazes on the HC foods. The number of initial direct gazes on an object reflects the degree of automatic attention to it (Garner et al., 2006). In this study, a higher number of initial gazes on the HC foods indicated greater automatic attention to them.

## Results

### Preliminary Analyses

There were significant differences between the S-REs and US-REs on the PSRS [*t*(62) = -9.69, *p* < 0.001], with the S-REs scoring higher (*M* = 4.55; *SD* = 0.55) than the US-REs (*M* = 3.21; *SD* = 0.55); thus, the classification of the two groups was reasonable. In addition, the BMI (*M* = 20.56, *SD* = 1.73 vs. *M* = 20.41, *SD* = 1.47), age (*M* = 20.77, *SD* = 1.41 vs. *M* = 21.15, *SD* = 1.15), hunger ratings (*M* = 0.51, *SD* = 0.29 vs. *M* = 0.44, *SD* = 0.26), food cravings (*M* = 0.28, *SD* = 0.28 vs. *M* = 0.39, *SD* = 0.30), and negative mood ratings (*M* = 0.17, *SD* = 0.18 vs. *M* = 0.17, *SD* = 0.18) of the S-REs and US-REs, respectively, were not significantly different (all *t*s < 1.49, all *p*s > 0.14).

The descriptive statistics of the main variables are presented in **Table 1**. Except for participants’ scores on the Restrained Eating subscale of the DEBQ [*t*(62) = -2.47, *p* = 0.02] and the PSRS [*t*(62) = -9.69, *p* < 0.001], no significant differences were found on the main variables between the S-REs and US-REs (all *t*s < -0.50, all *p*s > 0.15).

**Table 1:** Means and standard deviations on the measures of the main variables for the unsuccessful and successful restrained eaters.

	Unsuccessful restrained eaters		Successful restrained eaters	
	*M*	*SD*	*M*	*SD*
The number of initial direct gazes	52.83	8.59	53.97	7.58
Restraint eating	3.64	0.48	3.91	0.41
Perceived self-regulatory success in dieting	3.21	0.55	4.55	0.55
General inhibitory control	252.3	38.21	256.71	32.89
Food-specific inhibitory control	238.28	52.22	253.90	33.25
Food choices (%)	54.85	30.31	58.66	25.16

### Automatic Attention as a Predictor of Food Choice

To test whether IC moderated the association of automatic attention with food choice, we analyzed zero-order correlations between automatic attention, food choice, and inhibitory control (**Table 2**). Automatic attention to HC foods was positively correlated with the proportion of HC foods that were chosen (*r* = 0.41, *p* = 0.001). Participants’ HC food choices were not correlated with their PSRS, G-IC, or F-IC scores (all *r*s < 0.09, *p*s > 0.30).

**Table 2:** Zero order correlations for the main variables.

	1	2	3	4	5	6
1 The number of initial direct gazes	—					
2 Restraint eating	0.18 (0.17)	—				
3 Perceived self-regulatory success in dieting	0.10 (0.43)	0.32 (0.01)	—			
4 General inhibitory control	-0.13 (0.31)	-0.07 (0.62)	0.12 (0.38)	—		
5 Food-specific inhibitory control	0.14 (0.27)	-0.16 (0.23)	0.16 (0.23)	0.24 (0.06)	—	
6 Food choices (%)	0.41 (0.001)	-0.046 (0.73)	0.09 (0.47)	-0.13 (0.31)	-0.13 (0.31)	—

*The zero-order correlations are followed by their *p*-values, *r*(*p*).*

Furthermore, we performed a multiple regression analysis with the percentage of HC food choices selected as the dependent variable. We entered the PSRS, automatic attention, and IC (G-IC or F-IC) scores as the predictor variables. Next, we entered all possible two-way interaction terms for the PSRS, automatic attention, and IC (G-IC or F-IC) scores. Then, the three-way interaction terms were entered (Aiken et al., 1991; Hofmann et al., 2007; Wang et al., 2015).

**FIGURE 1:**
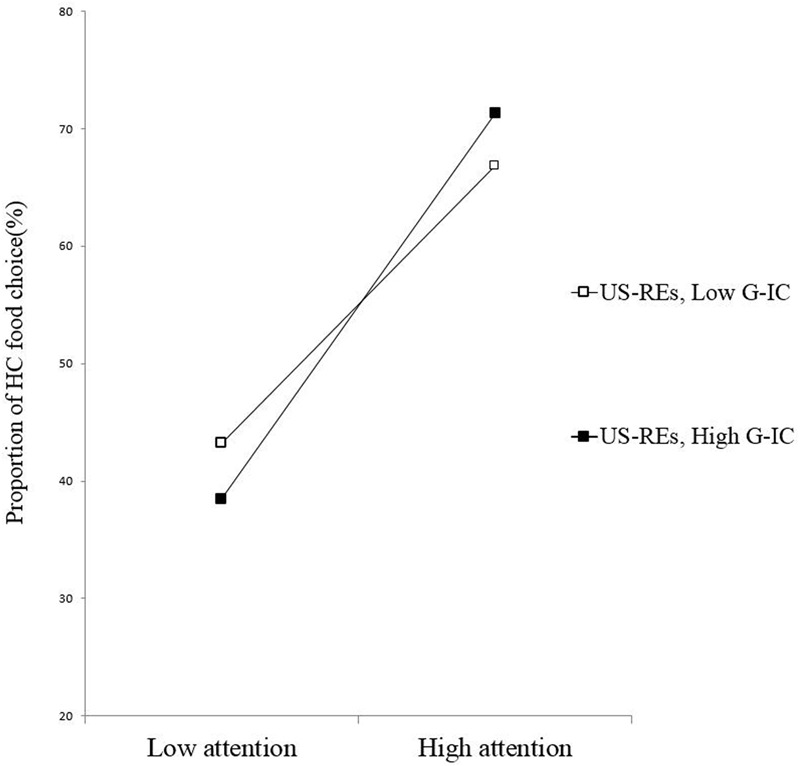
**Slopes for the automatic attention–food choice relationship across levels of G-IC among the US-REs**.

**FIGURE 2:**
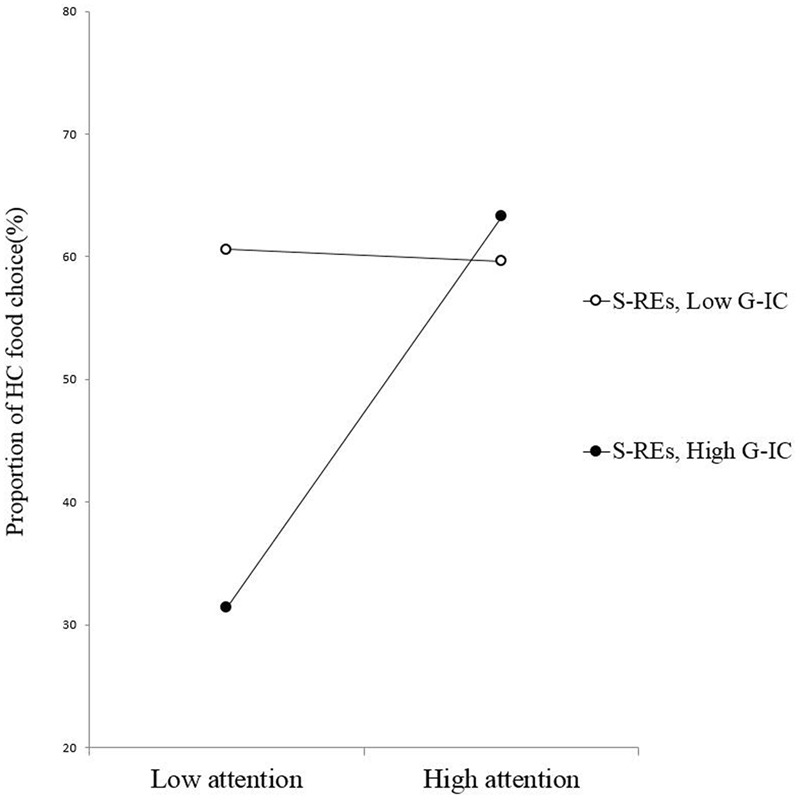
**Slopes for the automatic attention–food choice relationship across levels of G-IC among the S-REs**.

The results of the regression analysis (*R*^2^ = 0.25) with G-IC as the moderating variable, showed a three-way interaction of PSRS, automatic attention, and G-IC that was significant, β = -0.34, *p* = 0.02, power (1-β) = 0.97. Simple slope analyses revealed that among the US-REs (those with lower PSRS scores), automatic attention to the HC foods was positively related to the percentage of HC food choices for those with better G-IC (β = 0.63, *p* = 0.05), but not for those with poorer G-IC (β = 0.47, *p* = 0.10) (**Figure 1**). However, among the S-REs (those with higher PSRS scores), automatic attention to HC foods was not associated with the percentage of HC food choices, for either the participants with lower (β = 0.08, *p* = 0.81) or higher G-IC (β = 0.41, *p* = 0.16) (**Figure 2**).

**FIGURE 3:**
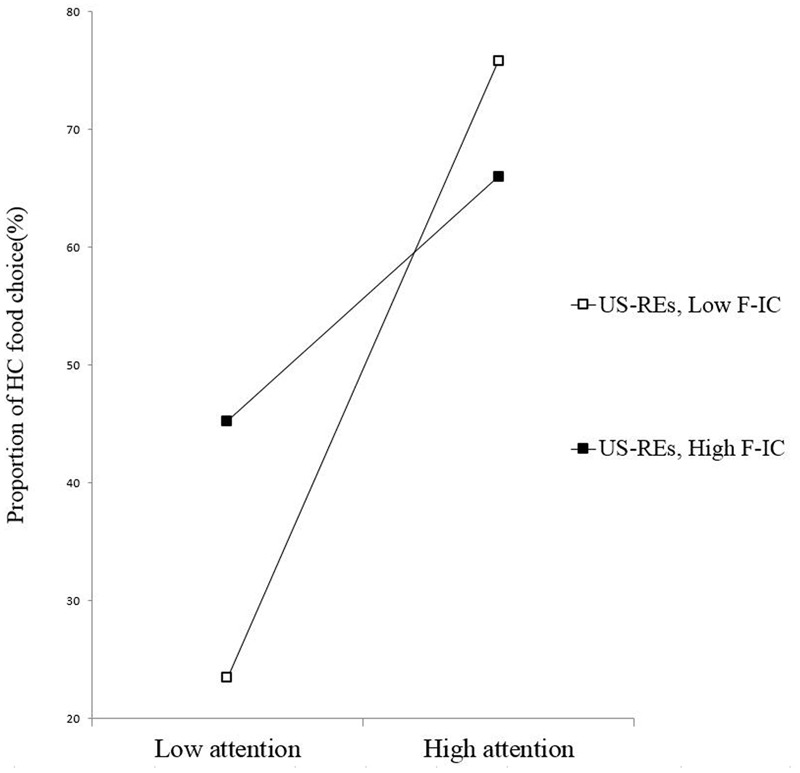
**Slopes for the automatic attention–food choice relationship across levels of F-IC among the US-REs**.

**FIGURE 4:**
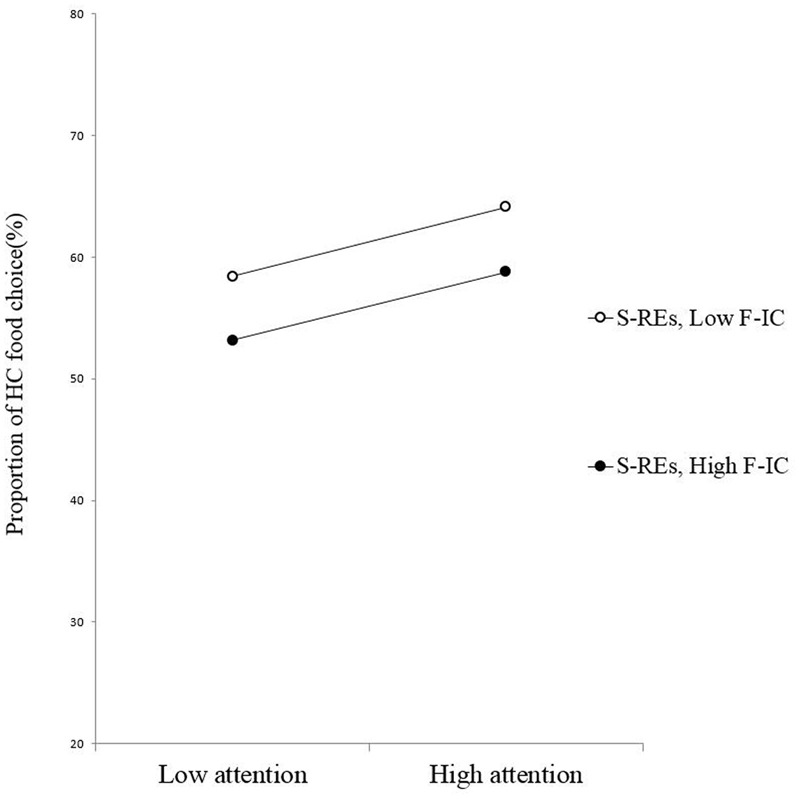
**Slopes for the automatic attention–food choice relationship across levels of F-IC among the S-REs**.

The results of the regression analysis (*R*^2^ = 0.30) with F-IC as the moderating variable, revealed a significant three-way interaction of PSRS, automatic attention, and F-IC, β = -0.54, *p* = 0.01, power (1-β) = 0.99. As confirmed by the simple slope test, among the US-REs (**Figure 3**), automatic attention to the HC foods was unrelated with the percentage of HC food choices when participants’ F-IC was better (β = 0.44, *p* = 0.12), but it was positively associated with the percentage of HC food choices when F-IC was poorer (β = 0.84, *p* = 0.003). For the S-REs (**Figure 4**), automatic attention to HC foods was uncorrelated with the percentage of HC food choices for those with either higher F-IC (β = 0.24, *p* = 0.44) or lower F-IC (β = 0.23, *p* = 0.35) scores.

## Discussion

To the best of our knowledge, the present study is the first to examine the relationship between impulsive systems and REs’ food choices and the moderating effects of G-IC and F-IC among S-REs and US-REs. We hypothesized that S-REs would not be affected by automatic attention (the impulsive system), regardless of whether their G-IC or F-IC were higher or lower. Lower G-IC or F-IC among the US-REs was expected to increase their automatic attention (the impulsive system) to their food choices. We found that the food choices of the REs were affected by the impulsive system (automatic attention), which was moderated by G-IC and F-IC. As expected, automatic attention of the S-REs to HC foods did not influence their food choices, regardless of whether their G-IC or F-IC was high or low. Unexpectedly, an effect of automatic attention on food choice was found among the US-REs with poor F-IC, but not those with poor G-IC.

Previous studies have reported a positive correlation between attentional bias and HC food consumption (Werthmann et al., 2011). Moreover, studies in which attentional bias was experimentally manipulated found that increased attention to food was accompanied by increased food consumption, whereas decreased attention to food was accompanied by decreased food consumption (Kemps et al., 2014; Werthmann et al., 2014). The results of the present study extends existing knowledge of the relationship between attention to food and food choices. We found that this relationship was moderated by G-IC and F-IC; however, most studies have focused on another component of the impulsive system, namely, implicit preferences for snack foods (Hofmann et al., 2009a; Wang et al., 2015) and the tendency to buy snacks on impulse (Honkanen et al., 2012). They have also found that G-IC (Hofmann et al., 2009a; Wang et al., 2015) and F-IC (Honkanen et al., 2012) moderated the relationship between the impulsive system and food consumption. Based on these studies’ findings, the premise has been accepted that the impulse system includes several types of mechanisms that affect eating behavior (including food consumption and food choice), especially the moderating effects of G-IC and F-IC on these relationships.

The most important finding of the present study was that the moderating effects of G-IC and F-IC differed among the US-REs. Among the US-REs with low F-IC, the impulsive system (automatic attention) had a stronger influence on eating behavior (food choice), whereas among those with low G-IC, the impulsive system did not influence eating behavior. The opposite moderating effect found for F-IC and G-IC, with poor F-IC (not poor G-IC) enhance the effect of impulsive system on food choice leading to unhealthy eating behaviors. However, the results were not surprising because many studies have reported an association between being overweight and inefficient F-IC, rather than G-IC (Nederkoorn et al., 2012; Houben et al., 2014). A recent investigation of the effects of F-IC and G-IC training on immediate snack food consumption found that the training effect involved only F-IC. When participants received F-IC training to inhibit their responses to pictures of food, their food intake decreased, but it did not decrease after G-IC training (Lawrence et al., 2015). These results showed that unhealthy dietary behaviors had a stronger association with poor F-IC, than with G-IC, which support the results of our study. Therefore, our results further demonstrate the premise that the roles of G-IC and F-IC of the impulsive system are different, with poor F-IC being the main cause of unhealthy eating among US-REs.

Conversely, the impulsive system in the present study was not associated with food choice, regardless of whether participants’ G-IC or F-IC were high or low among S-REs. The findings indicated that S-REs were less affected by the impulsive system than the US-REs, which might have been due to differences in the strength of the impulsive systems between the two groups. Previous studies have observed that when confronted with food temptations, S-REs paid less attention to HC foods (Zhang et al., 2016), automatically activated more concepts about dieting goals and weight management (Fishbach et al., 2003; Stroebe et al., 2013), use more flexible cognitive-control strategies (Meule et al., 2011), compare to US-REs. These findings might be accounted for by: (1) the S-REs’ impulsive systems, which are not stronger than those of US-REs, (2) S-REs’ dieting goals and flexible cognitive-control strategies inhibited the power of impulsive systems. These characteristics might have helped S-REs minimize the effects of the impulsive system on food choice, regardless of whether their G-IC or F-IC scores were high or low. In summary, US-REs exhibited greater automatic attention to food (impulsive system) and poor F-IC (not poor G-IC), which cause unsuccessful restrained eating, whereas S-REs effectively regulate their dietary intake.

This investigation of the impulsive systems of REs revealed that their effects on food choices depended on their G-IC and F-IC. In particular, only lower F-IC (not G-IC) heightened the effect of impulsive systems on food choices among the US-REs in our study. This finding suggests that the failure to restrain one’s eating is due to poor F-IC rather than poor G-IC. Consequently, the study provides constructive suggestions for clinical interventions, which should focus on F-IC training. In addition, low F-IC did not directly influence food choices; it played a moderating role by enhancing the effect of automatic attention to food choices. The US-REs with lower F-IC and greater automatic attention to HC foods chose HC foods more often. These results also suggest that interventions should not only focus on enhancing F-IC, but also on reducing automatic attention to HC foods. Finally, this study found that food choices were affected less by automatic attention, regardless of whether F-IC or G-IC were high or low among the S-REs. The differences in the mechanisms that were found among the S-REs and US-REs (S-REs were less affected by inhibitory control and automatic attention than US-REs), address the question of why some, but not all REs succeed.

This study has several limitations. First, the study sample only included individuals whose weight was within the normal range; therefore, a link between automatic attention, inhibitory control, and food choice in obesity cannot be inferred. Additionally, we cannot be sure of the role of the impulsive systems’ mechanisms in weight change. Further research is needed to compare people with a normal weight with those who are obese to identify the factors that induce weight gain, which should contribute to the development of interventions for obesity. Second, inhibitory control might fluctuate over time, similar to the state self-control. Actually, the trait of self-control, considered part of one’s personality, has been reported to be a consistent predictor of behavioral outcomes (Tangney et al., 2004; de Ridder et al., 2012). Few studies have focused on trait self-control. Thus, the relationship between the impulsive system, trait self-control, and food choice should be examined in future research. Finally, future studies should revise the food-choice task by designing food-choice situations with ecological validity to increase the generalizability of the results to real-world settings and to improve our effectiveness in detecting decision-making processes during the task. Doing so should also allow us to elucidate further the factors influencing restrained eating and thereby help people improve their diets.

## Author Contributions

XZ and SC designed the study and wrote the protocol. HC conducted literature searches and provided summaries of previous research studies. YG and WX conducted the statistical analysis. XZ wrote the first draft of the manuscript and all authors contributed to and have approved the final manuscript.

## Conflict of Interest Statement

The authors declare that the research was conducted in the absence of any commercial or financial relationships that could be construed as a potential conflict of interest.

## Abbreviations

F-IC: food-specific inhibitory control
G-IC: general inhibitory control
HC: high caloric
LC: low caloric
REs: restrained eaters
S-REs: successful restrained eaters
US-REs: unsuccessful restrained eaters
